# In Silico Screen Identifies a New Family of Agonists for the Bacterial Mechanosensitive Channel MscL

**DOI:** 10.3390/antibiotics11040433

**Published:** 2022-03-24

**Authors:** Robin Wray, Paul Blount, Junmei Wang, Irene Iscla

**Affiliations:** 1 Department of Physiology, University of Texas Southwestern Medical Center, 5323 Harry Hines Blvd., Dallas, TX 75390, USA; robin.wray@utsouthwestern.edu; 2 Computational Chemical Genomics Screening Center, Department of Pharmaceutical Sciences, School of Pharmacy, University of Pittsburgh, Pittsburg, PA 15261, USA

**Keywords:** bacterial channels, antibiotic resistance, bacterial drug target

## Abstract

MscL is a highly conserved mechanosensitive channel found in the majority of bacterial species, including pathogens. It functions as a biological emergency release valve, jettisoning solutes from the cytoplasm upon acute hypoosmotic stress. It opens the largest known gated pore and has been heralded as an antibacterial target. Although there are no known endogenous ligands, small compounds have recently been shown to specifically bind to and open the channel, leading to decreased cell growth and viability. Their binding site is at the cytoplasmic/membrane and subunit interfaces of the protein, which has been recently been proposed to play an essential role in channel gating. Here, we have targeted this pocket using in silico screening, resulting in the discovery of a new family of compounds, distinct from other known MscL-specific agonists. Our findings extended the study of this functional region, the progression of MscL as a viable drug target, and demonstrated the power of in silico screening for identifying and improving the design of MscL agonists.

## 1. Introduction

The mechanosensitive channel of large conductance, MscL, is a homopentameric protein found in the cytoplasmic membrane of the vast majority of bacterial species, as well as some fungi [1,2,3]. It serves as a biological emergency release valve, gated by membrane tension effected by the sudden decrease in the osmolarity of the environment [2,3,4]. The opening of MscL’s huge pore, on the order of 25 to 30 Å [5,6], allows osmoprotectants, which are accumulated or synthesized at higher osmolarity, to rapidly jettison from the cell, avoiding lyses and thus reaching osmotic homeostasis [3,7]. Not surprisingly, inappropriate gating of the MscL channel, either by mutations [8,9,10,11] or post-translational modifications [9], is detrimental to the cell, causing the channel to leak valuable metabolites, which leads to slowed growth and decreased viability.

These finding suggested that MscL could be a viable novel drug target for difficult-to-treat bacterial infections. Indeed, one study independently identified MscL as one of the top 20 potential bacterial drug targets [12]. Although amphipaths that add tension to the membrane have been known to gate bacterial mechanosensitive channels [3,13,14], these are non-specific, also gating an unrelated bacterial mechanosensitive channel, MscS (“S” for smaller conductance).

We have performed a high-throughput screen (HTS) [15] and identified two novel MscL agonists, with antibiotic and adjuvant properties [16,17,18]. Given their different structures, they surprisingly bind in a similar binding pocket, at the cytoplasmic membrane interphase in a region of dynamic protein–protein and protein–lipid interactions that are crucial for MscL gating [19,20,21].

One major hurdle is identifying additional candidate MscL agonists, especially those with a novel scaffold; these will act as leads for potential drugs that maintain MscL agonist specificity with greater efficacy. Here, we have used an in silico screen for compounds that bind the same binding site, specifically looking for compounds not related to those previously characterized. We have found a new family of related compounds that specifically activated MscL as their sole mechanism of action, experimentally showing all of the characteristics expected for agonists that bound to this region with a slightly different agonist characteristic. These findings demonstrated that in silico screening is a viable approach to identifying and improving the design of novel MscL agonists.

## 2. Results

### 2.1. In Silico Screening: The Identification and Characterization of a Small Family of Compounds That Bind at the Targeted Location

#### 2.1.1. An In Silico Screen Identifies a Small Family of Related Compounds from the ZINC Library That Are Candidates for Agonists for the *E. coli* MscL Channel

**Figure 1:**
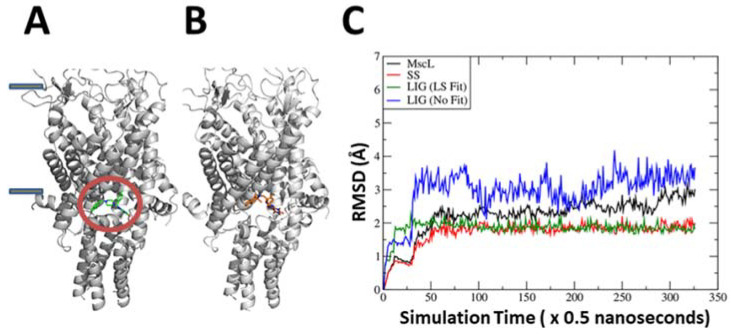
The binding site of compound 262 within the MscL structure as determined by docking and molecular dynamics (MD) simulations. (**A**) The docking pose of the compound, shown in green within the red circle at the cytoplasmic–membrane interface. The boundaries of the lipid bilayer are marked by the grey rectangles on the left. (**B**) Representative conformation of the ligand, in brown, within the pocket after MD simulation. Note that 262 resides in the binding pocket with little conformational change. (**C**) Root-mean-square deviation (RMSD) analysis over time shows that compound 262 underwent some translational or rotational movement (blue curve), but the overall conformations are very similar to the docking pose. “MscL” (black curve) is for all residues, while “SS” (red curve) is for residues in secondary structures. The green and blue curves represent the RMSDs of the ligand with and without least square fitting, respectively.

An in silico screen was performed with compounds from the ZINC library against the binding pocket previously found for the 011A and K05 MscL agonists. Promising hits were recognized according to their docking scores, and their agonist activities were confirmed by additional experimental approaches and more accurate molecular modeling techniques. We concentrated our efforts on “diverse” compounds not obviously related to those isolated previously. A small family of four related compounds appeared high on the list for having potential high affinity binding ZINC22855261, ZINC22409262, ZINC56952642, and ZINC22409190 (Supplementary Figure S1 shows the structures of these and previously characterized compounds that bind to this site). We will refer to the ZINC compounds by their unique last three identifying numbers (261, 262, 642, and 190). All members of this family were characterized using molecular dynamics (MD) simulations and in vivo growth and viability experiments. The binding free energy between each ligand and MscL was calculated using the MM–PBSA–WSAS approach for 300 snapshots collected from a 150 nanosecond MD trajectory (See Figure 1 and Supplementary Figures S2–S4). The compound 262 was the most stable during MD simulations and had the lower MM–PBSA–WSAS binding free energy, suggesting a higher affinity binding. Compound 262, with a lower binding affinity at −31.5 kcal/mol, was followed by compounds 261, 642, and 190 (Table S1). Given these data and the tractable nature of the molecule (see below), we concentrated our efforts on studying compound 262.

#### 2.1.2. Compound 262 Decreases *E. coli* Cell Growth and Viability in an MscL-Dependent Manner

**Figure 2:**
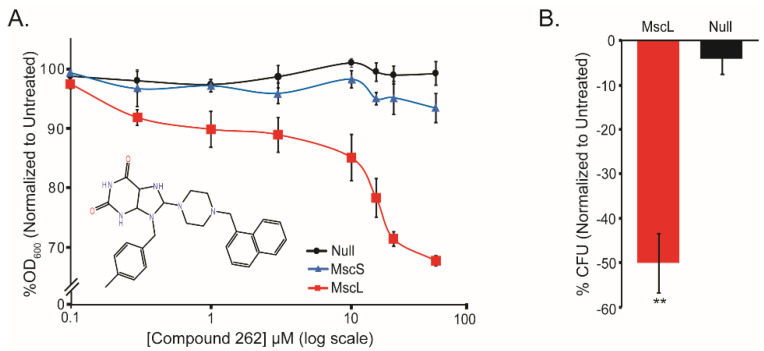
Compound 262 affects both the growth and viability of *E. coli* cultures in an MscL-dependent manner. *E. coli* MJF455 (ΔMscL and ΔMscS) bacterial strain are shown. For the expression of MscL (red) and MscS (blue), the pb10d expression vector (black) was used. (**A**) Growth inhibition in the presence of increasing concentrations of compound 262 is shown as the percentage of growth (OD_600_), normalized to untreated cultures. The structure of compound 262 is shown as an insert. Error bars show the standard error of the mean (SEM) (n = 3). (**B**) The percent reduction in CFU’s normalized to untreated samples after overnight treatment with 40 µM compound 262 (n = 4). ** *p* < 0.005 by a 2-tailed, homoscedastic *t*-test.

The compounds were then tested in vivo using whole-cell physiology. As predicted from the computational analyses, compound 262 decreased the growth and viability of bacterial cells in an MscL-dependent manner (Figure 2). In contrast, the expression of another bacterial mechanosensitive channel of a different family, MscS, did not make the strain susceptible to compound 262 (Figure 2A). We found that compounds 261 and 642 also decreased cell growth in an MscL-specific manner (Figure S7). However, compound 190 did not go into a 2% DMSO solution, demonstrating the limitations of the in silico screening approach and the necessity to complement this approach along with in vivo physiology. The ability of compound 262 to decrease growth and viability were similar to those previously observed for compounds 011A and K05 [16,17,18]; compound 262 showed a greater effect relative to compound 011A at similar concentrations (Figure S8), confirming the potential advantage of the in silico screening approach.

**Figure 3:**
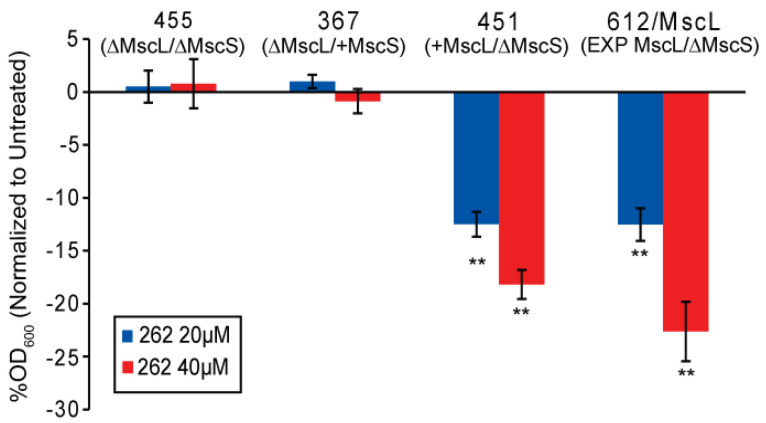
Compound 262 affects cultures with endogenous levels of expression of *E. coli* MscL. Cell lines used were indicated with endogenous levels of expression of MscL or MscL expressed in trans (EXP MscL), treated with compound 262 overnight at either 20 µM (blue) or 40 µM (red). The percent changes in OD_600_ normalized to untreated cultures are shown (n = 3). ** *p* < 0.005 by a 2-tailed, homoscedastic *t*-test when compared to MJF455 (ΔMscL and ΔMscS) at the same concentration.

The experimental data shown in Figure 2 were obtained from an MscL/MscS double-null strain with the channels, when expressed in trans. The expression construct used, pB10, is a mid-copy number plasmid that expresses MscL less than 10-fold greater than it is normally expressed endogenously [22]. However, even this relatively small increase in the expression of only a few folds could have consequences in terms of the results obtained. We therefore utilized strains selectively null for specific mechanosensitive channels and directly compared them to a strain expressing MscL in trans. As seen in Figure 3, two different concentrations of compound 262 were tested (20 and 40 µM), and the effects were similar to those obtained with MscL in trans; MscL-specific effects were observed at wild-type endogenous levels.

#### 2.1.3. MscL Is Sensitive to Compound 262 in Electrophysiological Experiments

**Figure 4:**
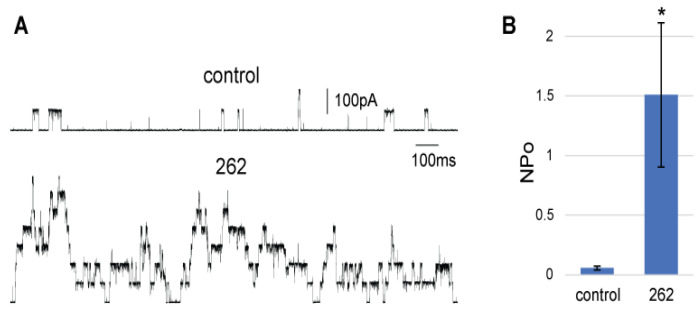
Compound 262 increases MscL activity in a patch clamp of native bacterial membranes. (**A**) Representative traces of MscL activity in an excised patch from bacterial giant cells, held at a pressure of −110 mmHg, before (control) and after 10 min of the addition of 40 μM compound 262 to the bath. (**B**) Quantification of MscL channel activity measured as the open probability (NPo) of the channels in a patch held at the same pressure, before and after curcumin treatment. * *p* < 0.05 Student paired *t*-test (n = 5).

We next tested if compound 262, like other agonists binding to the targeted site [16,17,18], increased the probability of opening (P_o_) of the MscL channel when assayed by a patch clamp of native membranes. As seen in Figure 4, MscL activity, measured in excised patches from giant spheroplasts [22], greatly increased with the addition of compound 262 in the bath chamber. A quantification of this effect is shown in Figure 4B; the probability of opening of MscL dramatically increased upon treatment with compound 262. It should be noted that in excised patches all cytoplasmic proteins were removed, so the compound was exposed only to the membrane and membrane proteins. The fact that we found that the compound worked in a bath with an inside-out excised patch, which correlated with addition of the compound to the cytoplasmic side, was also consistent with the other agonists that diffused across the membrane to obtain the binding site at the cytoplasmic–membrane interface (see Figure 1).

#### 2.1.4. Compound 262 Binds in the Targeted Pocket at the Cytoplasmic/Membrane and Subunit Interfaces

**Figure 5:**
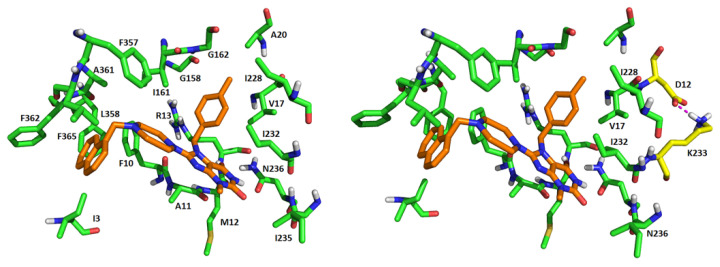
Two views of compound 262 in the binding pocket after MD simulations. Interactions with specific residues are shown.

Computational analyses of the binding of compound 262 to MscL were performed by MD simulations, leading to testable predictions by mutational analyses. The binding posture of the ligand and some of the potential interactions are shown in Figure 5; similar sites were found for the other compounds in the family (Figure S2).

**Figure 6:**
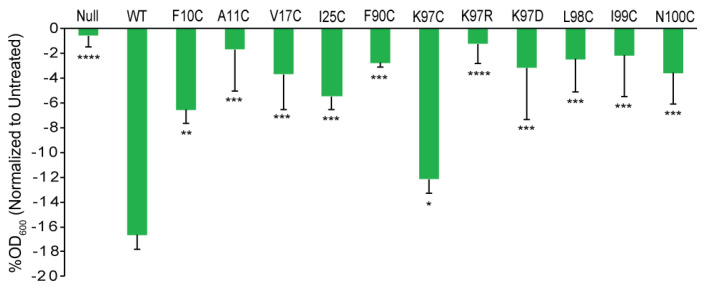
Mutational analysis provides additional evidence for the binding site of compound 262. The inhibition of growth after treatment of compound 262 at 40 µM is shown as OD_600_ normalized to untreated cultures. The MJF455 (ΔMscL and ΔMscS) cell lines carrying pB10d empty plasmid (Null) and expressing wild type *E. coli* MscL (WT) or mutations of *E. coli* MscL are indicated (n = 3–10). * *p* < 0.05, ** *p* < 0.005, *** *p* < 0.0005, **** *p* < 0.00005, mutations vs. WT for the *E. coli* MscL 2-tailed, homoscedastic *t*-test.

MscL residues crucial for its interaction with compound 262 were further identified by MM–GBSA analysis (ΔG values less than −1.2; see Table S2). These residues included F10, A11, V17, I25, I232 (I96), N236 (N100), and F362 (F90). In agreement with these predictions, as shown in Figure 6, these residues, when mutated, led to partial suppressors of the growth inhibition induced by compound 262 in WT MscL. Additionally, all mutations of residues K97, L98, and I99, known to contribute to the binding of other ligands to this pocket [16,17,18], also showed partial suppression. Hence, predictions from previous studies and computational analyses were supported by whole-cell physiological experiments.

**Figure 7:**
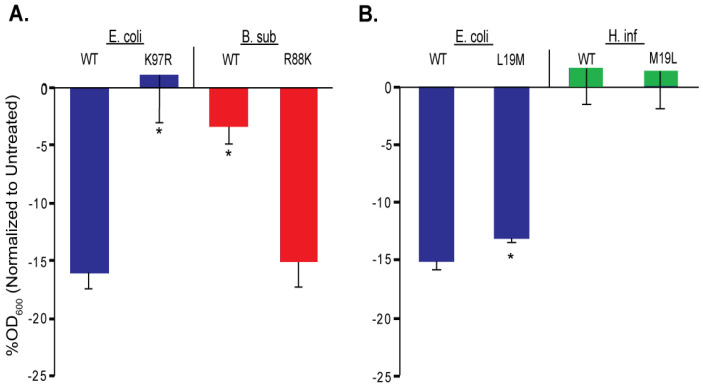
Compound 262 efficacies for cells expressing MscL orthologues and mutants of *Bacillus subtilis* and *Haemophilus influenza* (*H. inf*), which are consistent with its docking profile. *E. coli* strain MJF455 cultures was in the presence of 40 µM compound 262. (**A**) The percent changes in OD_600_ normalized to untreated cultures. Note that changing the *E. coli* MscL K97 to R decreased compound 262 sensitivity, whereas changing *B. sub*-MscL R88 to K increased compound 262 sensitivity, consistent with the canonical sequence in the proposed binding pocket. (**B**) *E. coli* expressing *E. coli* MscL (*E. coli*) or the orthologue *H. inf*. Note that changing the *E. coli* MscL L19 to M or *H. inf* M19 to L did not change compound 262 sensitivity, demonstrating compound 262 did not share the canonical binding pocket in the pore, where dihydrostreptomycin is known to bind. n = 3–6. * *p* < 0.05, mutations vs. WT for either *E. coli*-, *B. sub*-, or *H. inf*-MscL, 2-tailed, homoscedastic *t*-test.

Previously, to confirm binding sites, we have used orthologues that have differences in the proposed binding pocket. These orthologues showed a partial suppression of MscL-agonist-dependent slowed growth that is due to lower affinity binding. When the orthologues were modified to have a canonical binding site, an observed increase in agonist effectiveness gave strong evidence that the proposed site was indeed involved. In a general screen, we found that compound 262 was more effective against *E. coli* MscL than against *Bacillus subtilis* (Figure 7).

The *B. subtilis* MscL channel differed in the canonical binding site for 011A and K05, other agonists that bound in this location, and the channel can be modified to become sensitive to the agonists by mutating the site to the canonical binding site [16,17,18]. The primary residue that differed was the binding site K97, which is R in the analogous site in *B. subtilis* (R88). Consistent with previous results, we found that mutating this residue in *E. coli* to K97R led to a channel that did not respond well to compound 262 (Figure 6 and Figure 7). More significantly, the mutation of the analogous site in *B. subtilis* to the canonical site (R88K) led to an increased sensitivity to compound 262 (Figure 7A). These data strongly support that compound 262 bound to this site of the MscL-protein complex.

A second binding site, in the pore of the MscL channel, has been identified for dihydrostreptomycin. In previous studies, we have shown that this antibiotic binds to this site, opens the MscL channel pore and uses it as a pathway into the cytoplasm [23]. In this case, the canonical binding site was missing from the *Haemophilus influenza* (*H. inf*) orthologue, which can be mutated to make this orthologue sensitive to the antibiotic [23]. Although we found that *H. inf* was not sensitive to compound 262, the mutation that made MscL sensitive to dihydrostreptomycin did not make this homologue sensitive to compound 262 (Figure 7B), demonstrating the specificity to the previously described binding site for 011A and K05.

Together, the data were all consistent and confirmed that compound 262 binds to the MscL channel in the pocked used for the in silico screen, demonstrating the power of this approach to MscL-targeted drug development.

#### 2.1.5. Compound 262 Works Synergistically with Kanamycin and Other Antibiotics

**Figure 8:**
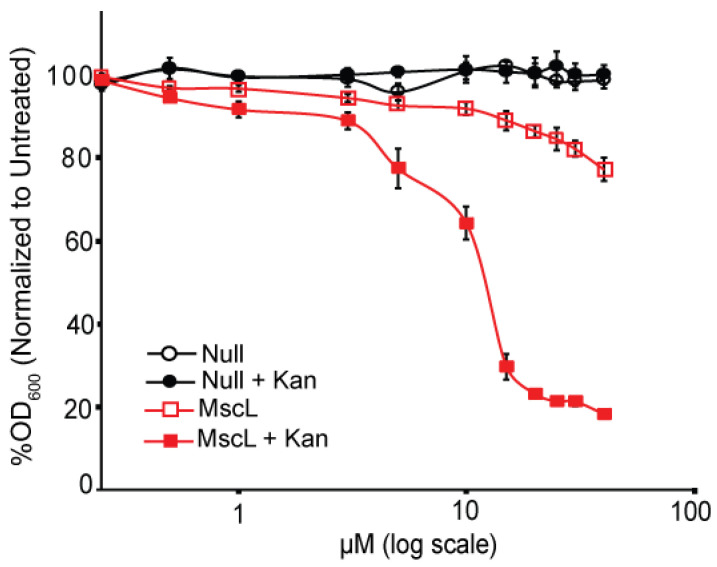
Compound 262 increases the potency of the common antibiotic kanamycin, only when MscL is present. Values are expressed as a percentage of growth (OD_600_), relative to non-treated samples. MJF455 (ΔMscL, ΔMscS) cultures carrying empty plasmid (Null) or expressing *E. coli* MscL (MscL) were treated with varying concentrations of compound 262 and grown in the presence or the absence of 0.5 μM kanamycin as indicated (n = 3–4). Error bars show the standard errors of the means (SEMs).

Previous experiments have demonstrated that MscL agonists work in concert with several other antibiotics by allowing them to passage into the cytoplasm [16,18,23]. As seen in Figure 8, at a sub-threshold concentration of kanamycin, the addition of compound 262 led to a decreased growth much greater than either compound alone. Similar results were obtained for tetracycline (Figure S9). These data are consistent with those obtained for other MscL agonists [16,18].

## 3. Discussion

Until the recent discovery of MscL-specific agonists in an HTS screen, [15,16,18,23], only compounds with amphipathic properties were known to activate bacterial mechanosensitive channels. These compounds, such as parabens and arachidonic acid, exert their unspecific effects mainly by altering the lipid membrane properties, to which many mechanosensitive channels prove sensitive (see [3] for a review).

Historically, one of the first chemical families used as topical antibiotics included dyes, some of which have been proposed to bind in the MscL pore [24]. An in silico screen to the region led to the discovery of “compound 10”, coined as Ramizol [24]. However, Ramizol appears to have a second mechanism of action. It also affects MscS to some degree, demonstrating that it is not entirely specific because, like many amphipaths, it adds tension to the membrane. Finally, the evidence for Ramizol binding to the MscL pore is limited to docking studies by computational analysis and has not yet been demonstrated experimentally by mutational analysis.

The original HTS screen we performed found a handful of known antibiotics whose potency was increased with MscL expression [15]; the best characterized of these antibiotics is dihydrostreptomycin, which has been shown by multiple approaches to directly bind to the pore of the MscL channel, activate it and pass through it into the bacterial cell cytoplasm, thus increasing its potency [23]. The observation that a handful of antibiotics were found in the screen [15] suggests that drugs having MscL agonist properties as one of their mechanisms or use MscL to get into the cell may play a role in several systems. Indeed, we recently found that MscL is gated by curcumin, a natural antibiotic compound found in turmeric, and it is one of the two major mechanisms of action behind the antibacterial properties of this drug [25]. It thus seems possible, if not likely, that MscL may underlie other dual-mechanism drugs where one of the mechanisms is membrane permeation and/or loss of membrane potential (e.g., see [26]); further investigation in this area is necessary.

The original screen, in addition to known antibiotics, identified several candidates for novel MscL agonists. Interestingly, the first two characterized (011A and K05), although not obviously structurally related (see Figure S1), shared a similar binding site. It is of interest to note that the binding site is at a subunit interface, a common feature for ligand-gated channels first found for the muscle acetylcholine channel [27]; it is also at the cytoplasm/membrane interface. The site of this binding pocket was not initially suspected for playing a large energetic contribution for MscL gating, because, unlike the pore, only a few mutations yielded gain-of-function phenotypes in random mutagenesis studies [8,10]. However, later, with the advance of our understanding of the functional role of N-terminal helix [28] and its interactions with the cytoplasmic regions of the TM domains [20], this region is now known to fine-tune large structural changes required for opening MscL pores [20,21].

Here, with the use of in silico screening, combined with MD simulations, we have successfully identified compounds that bind to this region that are new and distinct from those previously characterized, but sharing the property of MscL specificity. Interestingly, the family member best characterized in this manuscript, 262, was active in an HTS screen searching for compounds with antimicrobial activity against *M. tuberculosis* (PubChem AID 2842). This finding supports the notion that like 011A and K05, compound 262 is effective against other pathogens. This compound 262 candidate was never followed-up or published for its activity on *M. tuberculosis*, presumably because the researchers were looking for compounds inhibiting a tuberculosis-specific kinase (as indicated in the text)—compound 262 simply did not hit the desired target. We have now determined the target is MscL.

Compound 262 had many similarities to 011A and K05, the other agonists that bound to the same pocket. It inhibited growth and had some cidal activity. It worked at endogenous MscL expression levels. Electrophysiologically, it increased the probability of MscL channel opening in native membranes, even when the compound was added to the cytoplasmic side of the membrane. It also increased the potency of common antibiotics in an MscL-dependent manner, suggesting that while members of this class of compounds may not serve as stand-alone antibiotics, they may evolve into efficient adjuvants. Several approaches confirmed that it binds the targeted binding pocket shared by 011A and K05, but its interactions with some specific residues yield a unique pattern. The diversity of the compounds that bind to this generalized site in unique ways bodes well for isolating compounds with increased potency and efficacy; indeed, 262 was better in these properties than the first and best characterized MscL agonist, 011A (see Figure S4). Thus, the findings presented here demonstrated that in silico screening is a viable approach to identifying novel MscL agonists with advantageous characteristics.

In silico screening can enrich two types of libraries, the diverse and focused screening libraries. The former is suitable to identify novel hits with different structures, while the latter can optimize the existing hits and facilitate constructing structure–activity relationships. Prior to compound acquisition and bioassays, the top hits can be validated with more rigorous molecular modeling methods, e.g., a promising ligand can stably reside in a binding pocket during the MD simulations and has a decent binding affinity. Finally, the potency of the ligand is confirmed by bioassays. This approach was successfully applied here to identify promising activators of MscL using a gate-closed structure. However, there is a disadvantage of applying a closed MscL structure, i.e., one needs to run long MD simulations to conform if the ligand binding can trigger gate opening [18]. In addition, physiologically, because the compounds only destabilize the closed state, the channel opening is transient and the efficacy in vivo is low. On the other hand, it is more promising to apply an open structure in the above in silico HTS protocol to identify potent binding compounds that can lock the MscL channel into an open state. The calculated binding affinity should reflect the ability of a ligand to maintain the MscL in the open state, and this ability can be evaluated through short MD simulations. Toward this end, we have collected a set of open-state structures from MD simulations of the passage of dihydrostreptomycin and small proteins through the MscL channel [23]. In the future, utilizing established in silico HTS protocols on these open-channel structures should allow for the isolation of compounds that lock the channel in an open state and thus greatly increase the efficacy in bacterial cidal activity.

## 4. Materials and Methods

### 4.1. Strains and Cell Growth

The following bacterial *E. coli* Frag 1-derived strains were used as hosts in this study: MJF367 (ΔmscL::Cam), MJF451 (ΔMscS), MJF455 (ΔmscL::Cam and ΔMscS), and MJF612 (ΔmscL::cm, ΔmscS, ΔmscK::kan, and ΔybdG::aprΔ) [11,29]. The pB10d expression vector was used alone or with constructs inserted for expression; note that this was a mid-level expression vector and expressed MscL, for only a few times, endogenous levels. Cultures inoculated from a single colony were grown either in citrate-phosphate-defined media (CphM) (pH 7.0), consisting of the following per liter (8.57 g of Na_2_HPO_4_, 0.87 g of K_2_HPO_4_, 1.34 g of citric acid, 1.0 g NH_4_SO_4_, 0.001 g of thiamine, 0.1 g of MgSO_4_∙7H_2_O, and 0.002 g of (NH_4_)_2_SO_4_∙FeSO_4_∙H_2_O), incubated in a 37 °C shaker and rotated at 250 cycles per minute.

### 4.2. In Vivo Assays

#### 4.2.1. Growth Experiments

Growth inhibition was measured as previously described [15,23]. Briefly, overnight cultures of MJF376, MJF451, MJF455, and MJF612 strains carrying constructs were diluted at 1:50 (*v*:*v*) in CphM and grown, until an OD_600_ of 0.2 was reached. Expression was then induced by the addition of 1 mM isopropyl-b-D-thiogalactopyranoside (IPTG) for 30 min, 10 mM stocks of compound 262 solubilized in sterile dimethyl sulfoxide (DMSO) (Sigma-Aldrich, St. Louis, MO, USA) were diluted two times to achieve its final concentration in pre-warmed CphM with a final DMSO concentration of 2%, and 100 µL were added to wells of a pre-warmed, sterile 96-well flat bottom plate (Greiner bio-one, Monroe, NC, USA). Cultures were then diluted at 1:200 (*v*:*v*) in pre-warmed CphM, 100 µg/mL ampicillin, and 2 mM IPTG with diluted experimental antibiotics and were indicated, at 2× their concentration or mock (DMSO only). One hundred microliters of culture mixture with kanamycin A at a final concentrations of 0.2 µM (Sigma-Aldrich, St. Louis, MO, USA) were added to 96-well plates for a total of 200 µL, sealed with a sterile breathable film (Axygen, Union City, CA, USA), wrapped in aluminum foil and placed in a 37 °C shaker and rotated at 110 Cycles per minute for 16–17 h. OD_620_ was then taken with a Multiskan Ascent 354 (Thermo Fisher Scientific, Waltham, MA, USA) plate reader.

#### 4.2.2. Viability Experiments

Cultures from the above overnight growth experiments were used for all viability experiments performed as previously described [28]. Briefly, cultures were diluted at 1:20 (*v*:*v*) into pre-warmed CphM and serially diluted from 10^−3^ to 10^−6^ in a 96-well plate, and 5 µL liquid drops were placed on a pre-warmend LB ampicillin plates, which were placed in a 37 °C incubator. The next morning, colony-forming units were calculated to determine cell viability.

### 4.3. Electrophysiology

*E. coli* giant spheroplasts were generated and used for patch-clamp experiments as described previously (Blount 1999, trends microb). Excised, inside-out patches were examined at room temperature under symmetrical conditions using a buffer containing 200 mM KCl, 90 mM MgCl_2_, 10 mM CaCl_2_, and 5 mM HEPES (pH 6; Sigma-Aldrich, St. Louis, MO, USA). To study the effects of compound 262, each patch was measured before and after 10 min of the addition of the compound to the bath chamber, with the patch held at the same negative pressure. Recordings were performed at −20 mV (positive pipette). Data were acquired at a sampling rate of 20 kHz with a 5 kHz filter using an AxoPatch 200B amplifier in conjunction with Axoscope software (Axon Instruments, Union City, CA, USA). A piezoelectric pressure transducer (World Precision Instruments, Sarasota, FL, USA) was used to monitor the pressure throughout the experiments. Data were analyzed using Clampfit10 from pClamp10 software (Molecular Devices, San Jose, CA, USA).

### 4.4. Molecular Modeling and Computational Analyses

#### 4.4.1. In Silico Screenings

We performed in silico docking screening using a subset of the ZINC database that we compiled [30]. Each compound in this subset has one or more structurally similar drug molecules (Tanimoto coefficient: ≥0.85). All 123,192 compounds were ranked using the Glide [31] standard-precision docking scores. From this, 486 hits were identified using a docking score threshold of −9.5 kcal/mol. Then, 100 diverse compounds were selected for potential further studies. The Glide docking protocol was briefly described as follows: the binding site center was defined by a set of residues (I3, E6, F7, F10, I161, A361, F362, I364, F365, and K369), and the cubic binding site had a size length of 30 Å; the standard precision (SP) Glide was applied to evaluate docking poses with intramolecular hydrogen bonds being rewarded; the planarity of the conjugated pi groups was enhanced during the docking simulations; for each compound, up to 100 docking poses were minimized in the post-docking minimization step, and the best one was outputted. The same protocol was applied in our previous studies [16,17,18].

#### 4.4.2. MD Simulations and Free Energy Analysis

Compounds that had activities were further studied using more rigorous molecular modeling techniques. MD simulations were performed to study binding stability and to collect representative conformations for free energy analysis. Each ligand–MscL system contained one ligand, one MscL protein complex, 230 POPC lipids, 95 Cl^−^, 96 K^+^, and 32,312 TIP3P water molecules. The force filed models that describe the systems include FF14SB [32] for proteins, GAFF2 [33] for ligands, and LIPID14 [34] for POPC. The residue topologies of ligands were prepared using the Antechamber package [35]. Each system was first relaxed by a series of restrained minimizations with a constant harmonic restraint force applied to the main chain atoms decreased from 20 to 10, 5, and 1 kcal/mol/Å^2^ progressively. The system was then fully optimized without any restraint for 5000 steps. Next, 4-stage MD simulations were performed including the relaxation phase (four 100-picosecond MD simulations utilizing the same restraint scheme as minimizations), the system heating-up phase (five 100-picsecond MD simulations with the desirable temperature set to 50, 100, 150, 200, and 250 K), the equilibrium phase (298 K, 1 bar for 15 ns), and the final sampling phase (298 K, 1 bar for 150 ns). The integration of the equations of motion was conducted at a time step of 1 femtosecond (fs) for the first two phases and 2 fs for the last two phases. The root-mean-square deviations (RMSDs) of main chain atoms (for MscL) and heavy atoms (for ligands) during MD simulation time were calculated to investigate the conformational changes upon ligand binding. Two types of RMSDs were calculated for a ligand: LS-Fit describes the conformational change of the ligand, while No-Fit also accounts for the translational and rotational movement of the ligand inside the binding pocket. No-Fit RMSDs were directly calculated, after MD snapshots were aligned to the reference structure (the initial model of MscL) using secondary structures only. The MD conformation, which had the smallest RMSDs to the average structure of all the collected MD snapshots, was identified as the representative MD conformation of an MD system. Besides the RMSD, we also evaluated the fluctuations of MscL and the overall size of the simulations system during MD simulations. The B-factors of individual residues and the radius of gyration (RoG) were shown in Figures S5 and S6, respectively. Overall, the B-factor and RoG data are reasonable and consistent with the RMSD result.

All 3000 snapshots sampled from the last phase were used to conduct the MM–GBSA binding free energy decomposition analysis, while only 150 evenly selected snapshots were subjected to MM–PBSA–WSAS binding free energy calculation. For the MM–GBSA free energy decomposition, the internal dielectric constant was set to 1, while the external dielectric constant was set to two for modeling the hydrophobic part of the POPC lipids. Unlike the MM–GBSA free energy decomposition, we applied two external dielectrics (ε_wat_ = 80 for water and ε_lip_ = 2.0 for the lipid bilayer) in the MM–PBSA–WSAS binding free energy calculations. The membrane center offset parameter, mctrdz, which is defined as the distance between the coordinate center of all phosphorous atoms and the coordinate center of MscL/ligand complex, and the thickness of membrane, mthick, which is defined as the distance between the coordinate centers of the phosphorous atoms in the upper and lower layers, varied from one MD snapshot to another. Thus, those two parameters were calculated for each individual snapshot. For the ligand itself, the implicit membrane option was turned off, and the external dielectric constant was set to 80. The nonpolar part of a solvation free energy was calculated using the following equation: $\Delta G_{nonpolar}=0.0054\times \mathrm{SAS}+0.92$, where SAS is the solvent accessible surface area and 0.0054 is the surface tension coefficient parameter. The entropic term was estimated using the WSAS method described [36]. All the MD simulations and the free energy analyses were performed using the AMBER18 software package [37].

## Data Availability

Not applicable.

## Supplementary Materials

The following supporting information can be downloaded at: https://www.mdpi.com/article/10.3390/antibiotics11040433/s1, Figure S1: Structures of MscL-specific agonists that bind within the same region of the channel; Figure S2: Computational analyses of compound 261; Figure S3: Computational analyses of compound 642; Figure S4: Computational analyses of compound 190; Figure S5: Residue-based B-factor for the MD trajectories; Figure S6: The time courses of radius of gyration; Figure S7: Two closely related compounds also inhibit growth of *E. coli* cultures in an MscL-dependent manner; Figure S8: Comparison of the effects of two MscL agonists on *E. coli* growth; Figure S9: Compound 262 can also increase the potency of the common antibiotic tetracycline, when MscL is present; Table S1: MM–PBSA–WSAS binding free energy of the identified activators of the *E. coli* MscL channel; Table S2: MM–GBSA ligand–residue Interaction energies.

## References

[1] Booth I.R., Blount P. (2012). The MscS and MscL families of mechanosensitive channels act as microbial emergency release valves. J. Bacteriol..

[2] Cox C.D., Bavi N., Martinac B. (2018). Bacterial Mechanosensors. Annu. Rev. Physiol..

[3] Blount P., Iscla I. (2020). Life with Bacterial Mechanosensitive Channels, from Discovery to Physiology to Pharmacological Target. Microbiol. Mol. Biol. Rev..

[4] Levina N., Totemeyer S., Stokes N.R., Louis P., Jones M.A., Booth I.R. (1999). Protection of *Escherichia coli* cells against extreme turgor by activation of MscS and MscL mechanosensitive channels: Identification of genes required for MscS activity. EMBO J..

[5] Cruickshank C.C., Minchin R.F., Le Dain A.C., Martinac B. (1997). Estimation of the pore size of the large-conductance mechanosensitive ion channel of *Escherichia coli*. Biophys. J..

[6] Perozo E., Cortes D.M., Sompornpisut P., Kloda A., Martinac B. (2002). Open channel structure of MscL and the gating mechanism of mechanosensitive channels. Nature.

[7] Poolman B., Blount P., Folgering J.H., Friesen R.H., Moe P.C., van der Heide T. (2002). How do membrane proteins sense water stress?. Mol. Microbiol..

[8] Maurer J.A., Dougherty D.A. (2001). A high-throughput screen for MscL channel activity and mutational phenotyping. Biochim. Biophys. Acta.

[9] Iscla I., Wray R., Eaton C., Blount P. (2015). Scanning MscL Channels with Targeted Post-Translational Modifications for Functional Alterations. PLoS ONE.

[10] Ou X., Blount P., Hoffman R.J., Kung C. (1998). One face of a transmembrane helix is crucial in mechanosensitive channel gating. Proc. Natl. Acad. Sci. USA.

[11] Levin G., Blount P. (2004). Cysteine scanning of MscL transmembrane domains reveals residues critical for mechanosensitive channel gating. Biophys. J..

[12] Barh D., Jain N., Tiwari S., Parida B.P., D’Afonseca V., Li L., Ali A., Santos A.R., Guimarães L.C., de Castro Soares S. (2011). A novel comparative genomics analysis for common drug and vaccine targets in *Corynebacterium pseudotuberculosis* and other CMN group of human pathogens. Chem. Biol. Drug. Des..

[13] Martinac B., Adler J., Kung C. (1990). Mechanosensitive ion channels of *E. coli* activated by amphipaths. Nature.

[14] Perozo E., Kloda A., Cortes D.M., Martinac B. (2002). Physical principles underlying the transduction of bilayer deformation forces during mechanosensitive channel gating. Nat. Struct. Biol..

[15] Iscla I., Wray R., Wei S., Posner B., Blount P. (2014). Streptomycin potency is dependent on MscL channel expression. Nat. Commun..

[16] Wray R., Herrera N., Iscla I., Wang J., Blount P. (2019). An agonist of the MscL channel affects multiple bacterial species and increases membrane permeability and potency of common antibiotics. Mol. Microbiol..

[17] Wray R., Iscla I., Kovacs Z., Wang J., Blount P. (2019). Novel compounds that specifically bind and modulate MscL: Insights into channel gating mechanisms. FASEB J..

[18] Wray R., Wang J., Iscla I., Blount P. (2020). Novel MscL agonists that allow multiple antibiotics cytoplasmic access activate the channel through a common binding site. PLoS ONE.

[19] Iscla I., Wray R., Blount P. (2011). An in vivo screen reveals protein-lipid interactions crucial for gating a mechanosensitive channel. FASEB J..

[20] Iscla I., Wray R., Blount P. (2012). The dynamics of protein-protein interactions between domains of MscL at the cytoplasmic-lipid interface. Channels.

[21] Li J., Guo J., Ou X., Zhang M., Li Y., Liu Z. (2015). Mechanical coupling of the multiple structural elements of the large-conductance mechanosensitive channel during expansion. Proc. Natl. Acad. Sci. USA.

[22] Blount P., Sukharev S.I., Moe P.C., Martinac B., Kung C., Conn P.M., Conn P.M. (1999). Mechanosensitive channels of bacteria. Methods in Enzymology.

[23] Wray R., Iscla I., Gao Y., Li H., Wang J., Blount P. (2016). Dihydrostreptomycin Directly Binds to, Modulates, and Passes through the MscL Channel Pore. PLoS Biol..

[24] Iscla I., Wray R., Blount P., Larkins-Ford J., Conery A.L., Ausubel F.M., Ramu S., Kavanagh A., Huang J.X., Blaskovich M.A. (2015). A new antibiotic with potent activity targets MscL. J. Antibiot..

[25] Wray R., Iscla I., Blount P. (2021). Curcumin activation of a bacterial mechanosensitive channel underlies its membrane permeability and adjuvant properties. PLoS Pathog..

[26] Martin J.K., Sheehan J.P., Bratton B.P., Moore G.M., Mateus A., Li S.H., Kim H., Rabinowitz J.D., Typas A., Savitski M.M. (2020). A Dual-Mechanism Antibiotic Kills Gram-Negative Bacteria and Avoids Drug Resistance. Cell.

[27] Blount P., Merlie J.P. (1989). Molecular basis of the two nonequivalent ligand binding sites of the muscle nicotinic acetylcholine receptor. Neuron.

[28] Iscla I., Wray R., Blount P. (2008). On the structure of the N-terminal domain of the MscL channel: Helical bundle or membrane interface. Biophys. J..

[29] Schumann U., Edwards M.D., Rasmussen T., Bartlett W., van West P., Booth I.R. (2010). YbdG in Escherichia coli is a threshold-setting mechanosensitive channel with MscM activity. Proc. Natl. Acad. Sci. USA.

[30] Wang J., Ge Y., Xie X.Q. (2019). Development and Testing of Druglike Screening Libraries. J. Chem. Inf. Model.

[31] Friesner R.A., Banks J.L., Murphy R.B., Halgren T.A., Klicic J.J., Mainz D.T., Repasky M.P., Knoll E.H., Shelley M., Perry J.K. (2004). Glide: A new approach for rapid, accurate docking and scoring. 1. Method and assessment of docking accuracy. J. Med. Chem..

[32] Maier J.A., Martinez C., Kasavajhala K., Wickstrom L., Hauser K.E., Simmerling C. (2015). ff14SB: Improving the Accuracy of Protein Side Chain and Backbone Parameters from ff99SB. J. Chem. Theory Comput..

[33] Wang J., Wolf R.M., Caldwell J.W., Kollman P.A., Case D.A. (2004). Development and testing of a general amber force field. J. Comput. Chem..

[34] Dickson C.J., Madej B.D., Skjevik A.A., Betz R.M., Teigen K., Gould I.R., Walker R.C. (2014). Lipid14: The Amber Lipid Force Field. J. Chem. Theory Comput..

[35] Wang J., Wang W., Kollman P.A., Case D.A. (2006). Automatic atom type and bond type perception in molecular mechanical calculations. J. Mol. Graph. Model..

[36] Wang J., Hou T. (2012). Develop and test a solvent accessible surface area-based model in conformational entropy calculations. J. Chem. Inf. Model.

[37] Case D.A., Berryman J.T., Betz R.M., Cerutti D.S., Cheatham T.E., Darden T.A., Duke R.E., Giese T.J., Gohlke H., Goetz A.W. Amber 2018. www.ambermd.org.

