# Supplementary material for: In Silico Screen Identifies a New Family of Agonists for the Bacterial Mechanosensitive Channel MscL
Source: Antibiotics (Basel). 2022 Mar 24;11(4):433. doi: 10.3390/antibiotics11040433 (PMC9030384; doi:10.3390/antibiotics11040433)
Supplement: Supplementary file 1 [file antibiotics-11-00433-s001.zip › antibiotics-1628176-supplementary.pdf]

Supplementary Materials

In silico screen identifies a new family of agonists for the bacterial mechanosensitive channel MscL

Robin Wray, Paul Blount, Junmei Wang and Irene Iscla

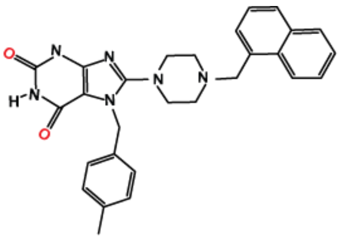

Compound 262

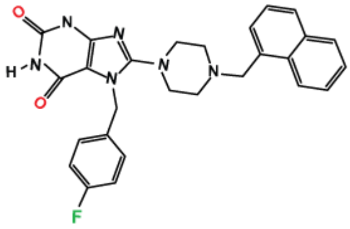

Compound 261

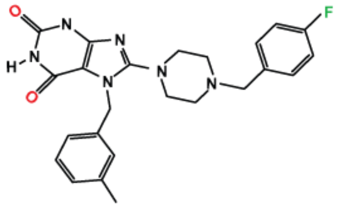

Compound 642

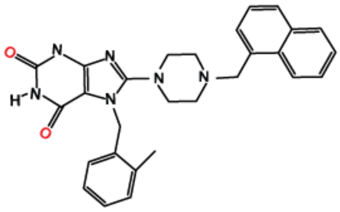

Compound 190

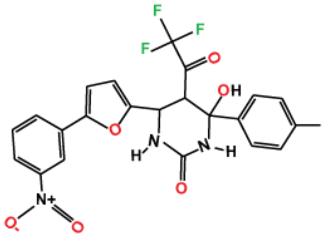

Compound K05

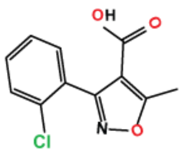

Compound 011A

Figure S1. Structures of MscL-specific agonists that bind within the same region of the channel. The top three and bottom-left compounds are members of a family found within the *in silico* screen described here; all are active with the exception of compound 190, which is insoluble; the K05 and 011A compounds were found in a traditional HTS and have been previously described, as discussed in text.

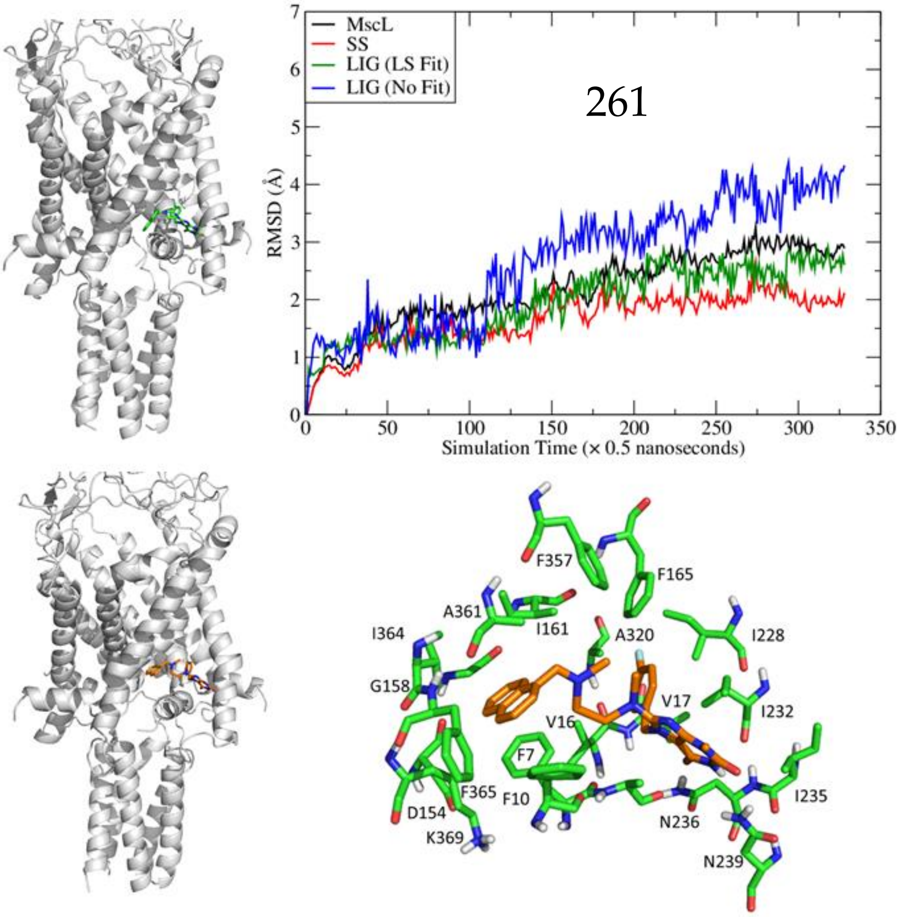

Figure S2. Computational analyses of compound 261. The top left of the quadrangle shows: the docking pose of the compound, shown in green at the cytoplasmic-membrane interface; the bottom left shows a representative conformation of the ligand, in brown, within the pocket after MD simulation. The RMSD analysis over time (top right) and the binding pocket after MD simulations with interactions with specific residues (bottom right) are shown.

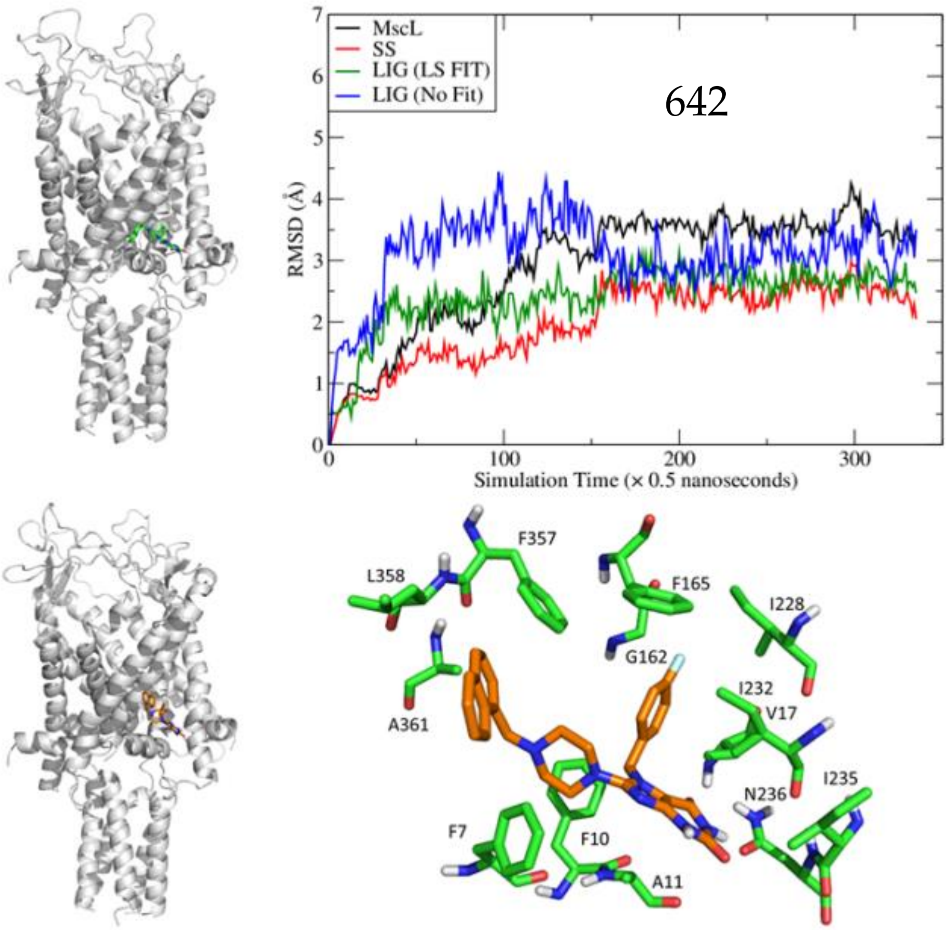

Figure S3. Computational analyses of compound 642. The top left of the quadrangle shows: the docking pose of the compound, shown in green at the cytoplasmic-membrane interface; the bottom left shows a representative conformation of the ligand, in brown, within the pocket after MD simulation. The RMSD analysis over time (top right) and the binding pocket after MD simulations with interactions with specific residues (bottom right) are shown.

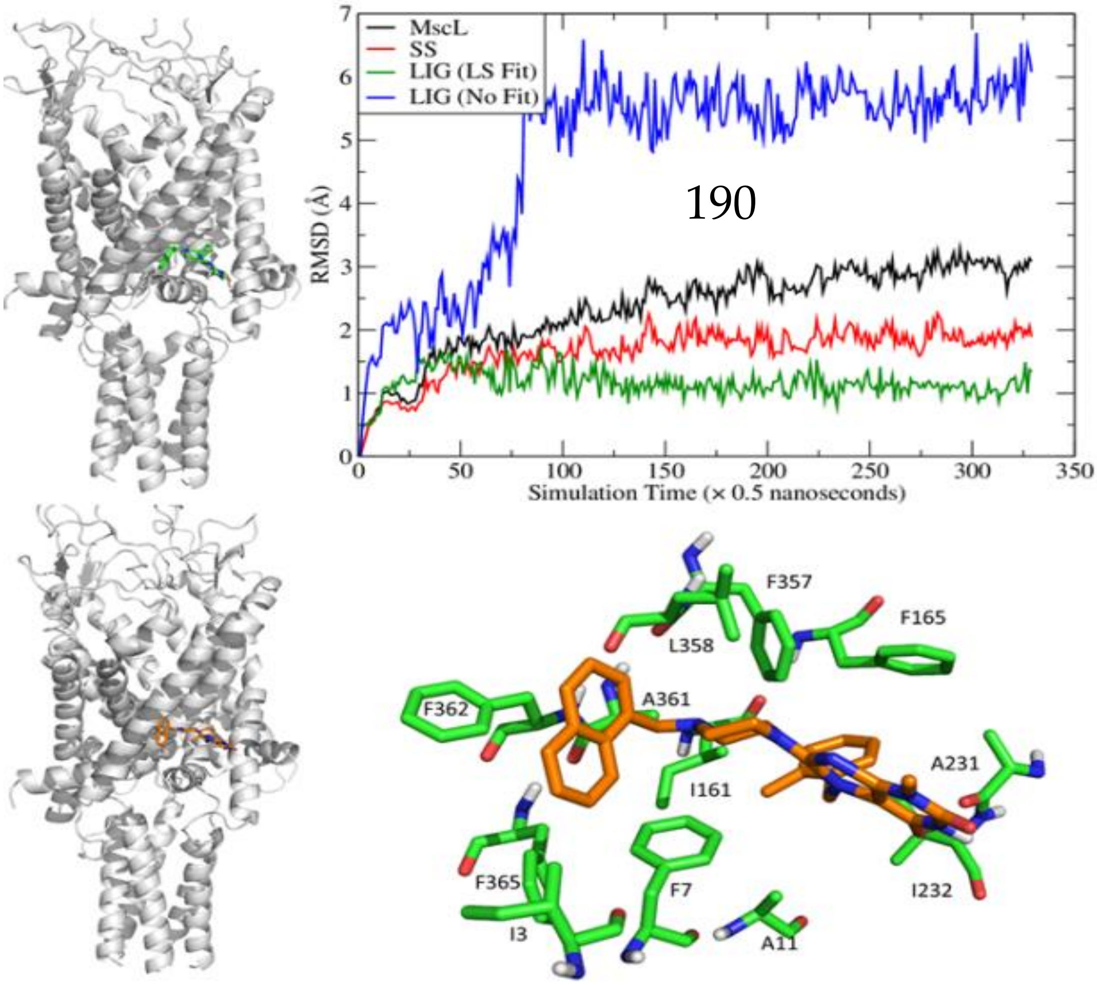

Figure S4. Computational analyses of compound 190. The top left of the quadrangle shows: the docking pose of the compound, shown in green at the cytoplasmic-membrane interface; the bottom left shows a representative conformation of the ligand, in brown, within the pocket after MD simulation. The RMSD analysis over time (top right) and the binding pocket after MD simulations with interactions with specific residues (bottom right) are shown.

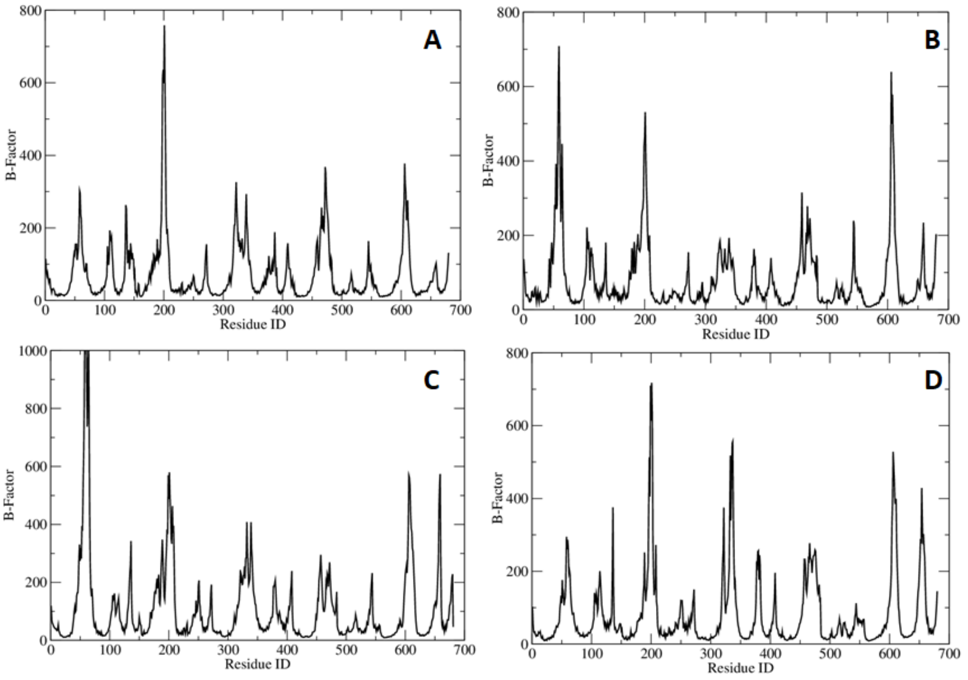

Figure S5. Residue-based B-factor calculated from MD trajectories for the four Ec-MscL/Ligand systems: A 262, B: 261, C: 642, D: 190. Periplasmic loops (Res IDs 47-77, 183-213, 319-349, 455-485, 591-621) and loops between transmembrane helices (TMs) and cytoplasmic helical bundle (Res IDs 103-118, 239-254, 375-390, 511-526,647-662) apparently have larger B-Factors.

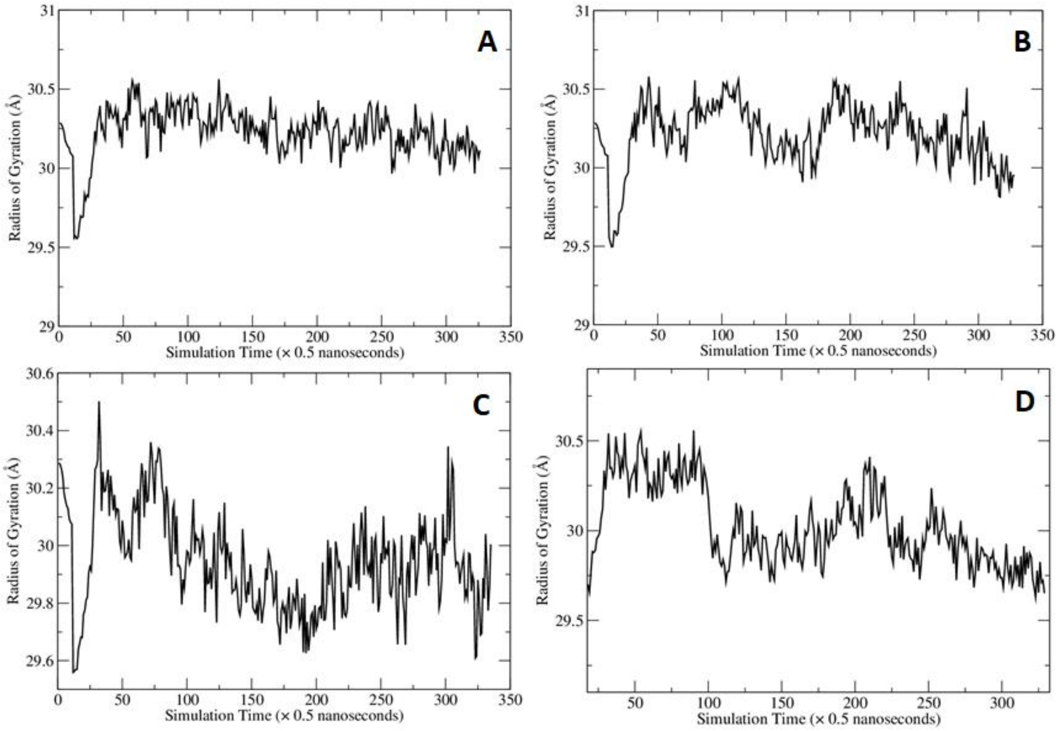

Figure S6. The time course of radius of gyration (RoG) for the four Ec-MscL/Ligand systems: A 262, B: 261, C: 642, D: 190. It is shown that all the systems have fluctuations between 29.5 and 30.5 Å and equilibrium was reached after the equilibrium phase.

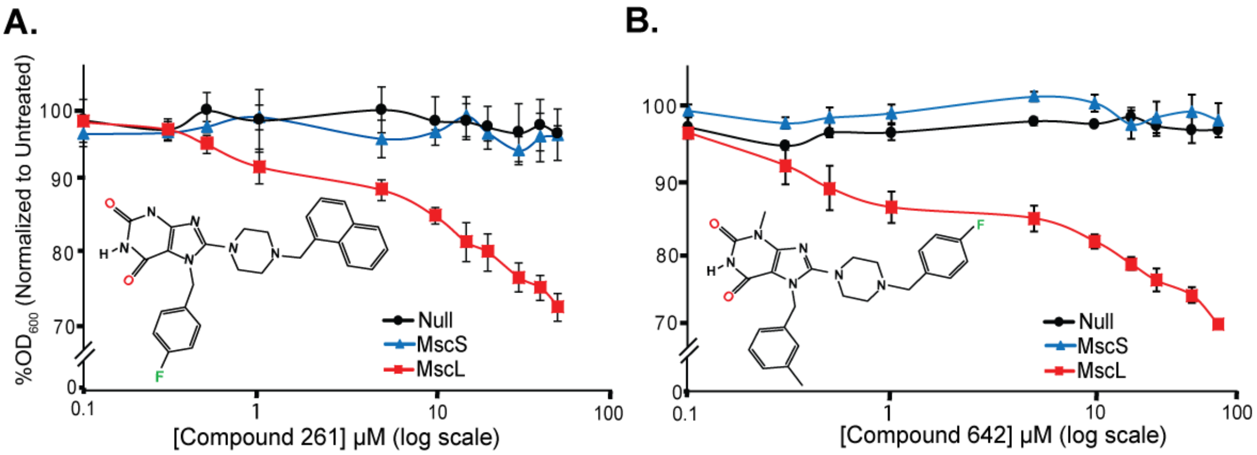

Figure S7. Two closely related compounds also inhibit growth of *E. coli* cultures in a MscL dependent manner. Shown is the *E. coli* strain MJF455 (ΔMscL, ΔMscS) carrying vector only (black circles) or expressing MscS (blue diamonds) or MscL (red squares). A) Percentage of decreased growth after treatment with compound 261 relative to untreated samples. The structure of compound 261 is shown as an insert. Note that the only change from 262 is an additional fluorine. (n=3) (B) Percentage of decreased growth after treatment with compound 642 relative to untreated samples. The structure of compound 642 is shown as an insert (n=3). All error bars show standard error of the mean (SEM).

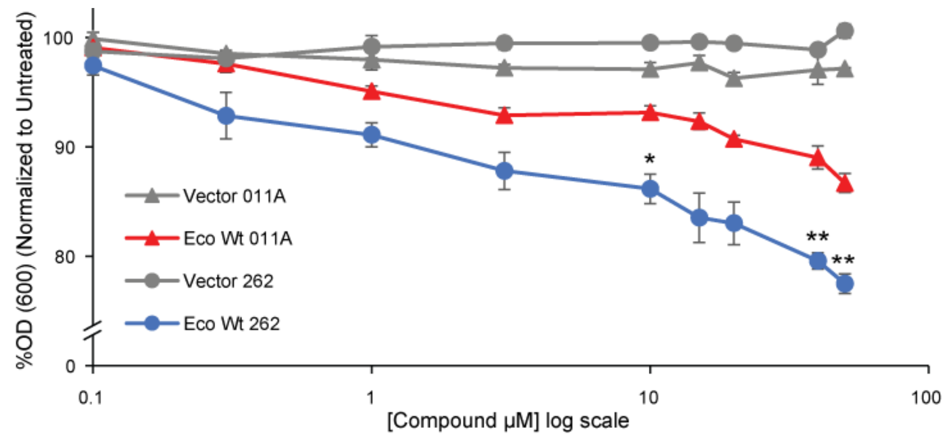

Figure S8. Comparison of the effects of two MscL agonists on E.coli growth. Cultures of E.coli MJF455 cells (ΔMscL, ΔMscS) carrying an empty vector or expressing MscL, were treated with increasing concentrations of MscL agonist 011A (triangles), or compound 262 (circles). In both cases the effect proved to be MscL specific, but the potency and efficacy of 262 is significantly higher that 011A. *p<0.05, **p<0.003 t-test unpaired Eco MscL treated with 011A vs 262 (n=3).

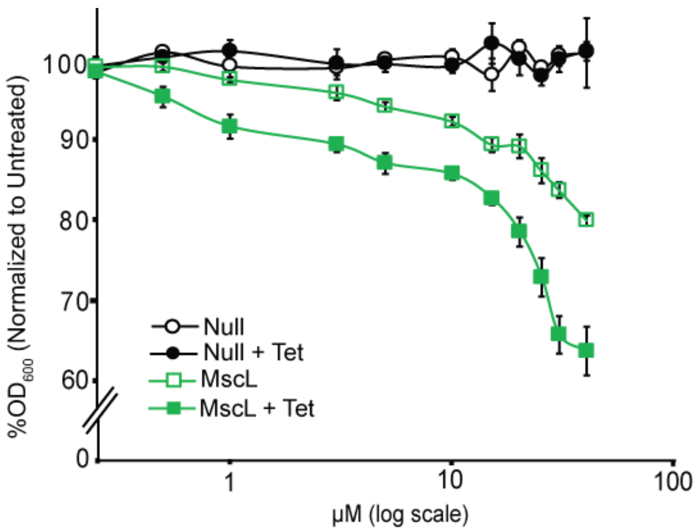

Figure S9. Compound 262 can also increase the potency of the common antibiotic tetracycline when MscL is present. A final concentrations of at 0.25 µM Tetracycline Hydrochloride (Thermo Fisher Scientific Waltham, MA) in 100 µl of culture mixture was added to the 96 will plates for a total of 200 µl, sealed with a sterile breathable film (Axygen, Union City, CA), wrapped in aluminum foil and placed in a 37°C shaker, rotated at 110 Cycles per minute for 16-17 hours and OD_{620} was then taken with a Multiskan Ascent 354 (Thermo Fisher Scientific Waltham, MA) plate reader. Values are expressed as a percentage of growth (OD_{600}), relative to non-treated samples. MJF455 (ΔMscL, ΔMscS) cultures carrying empty plasmid (Null) or expressing *E. coli*-MscL (MscL), treated with varying concentrations of compound 262, grown in the presence or the absence of tetracycline as indicated (n=4). error bars show standard error of the mean (SEM).

Table S1. MM-PBSA-WSAS Binding Free Energy of the Identified activators of E. coli MscL Channel. All Energy Terms Are in kcal/mol.

Compound	ΔE_{VDW}	ΔE_{eel}	ΔG_{sol}^{polar}	$\Delta G_{sol}^{nonpolar}$	TΔS	ΔG_{MMPBSA}
262	-49.77 ± 0.42	-17.72 ± 0.21	16.78 ± 0.05	-4.54 ± 0.02	-22.40 ± 0.07	-32.86 ± 0.32
261	-53.73 ± 0.12	-9.87 ± 0.19	17.27 ± 0.32	-4.89 ± 0.02	-23.94 ± 0.07	-27.29 ± 0.35
190	-35.95 ± 0.32	-6.27 ± 0.26	16.20 ± 0.02	-3.55 ± 0.04	-18.54 ± 0.07	-11.04 ± 0.26
642	-44.89 ± 0.24	-13.77 ± 0.49	17.00 ± 0.03	-4.20 ± 0.01	-21.48 ± 0.10	-24.37 ± 0.28

Table S2. MM-GBSA Ligand-Residue Interaction Energies. All Energy Terms Are in kcal/mol. All interaction energies better than -2.0 kcal/mol were colored in red and those between -2.0 and -1.0 kcal/mol were colored in blue.

Res Type	Res ID	Res ID *	$\Delta G_{Ligand\text{-}Residue}$ (kcal/mol)			
			Compound 262	Compound 261	Compound 190	Compound 642
ILE	3	3	-0.38	-0.28	-0.41	-
ILE	4	4	-	-	-0.19	-
GLU	6	6	-0.14	-0.56	-	-0.21
PHE	7	7	-0.24	**-1.85**	**-2.16**	**-2.10**
ARG	8	8	-0.11	-0.15	-0.43	-
GLU	9	9	-	-0.30	-	-0.16
PHE	10	10	**-2.29**	**-2.46**	-0.23	**-1.49**
ALA	11	11	**-2.02**	**-1.64**	-0.55	**-1.94**
MET	12	12	-0.85	-0.17	-	-0.18
ARG	13	13	**-1.49**	-	-	-
VAL	16	16	-0.67	-0.90	-0.15	-0.53
VAL	17	17	**-1.27**	**-1.23**	-0.25	**-1.24**
ALA	20	20	-0.31	-0.39	-	-0.24
VAL	21	21	-	-0.16	-	-
ILE	24	24	-	-0.13	-	-
ASP	154	18	-0.15	-0.67	-	-0.16
LEU	155	19	-	-0.21	-	-
GLY	158	22	-0.48	-0.24	-	-0.11
VAL	159	23	-0.17	-	-	-
ILE	161	25	**-1.35**	-0.98	-0.74	-0.90
GLY	162	26	-0.36	-0.11	-0.13	-0.44
PHE	165	29	-0.16	-0.61	-0.88	**-1.22**
ILE	228	92	-0.39	-0.37	-	-0.37
ALA	231	95	-	-	-0.29	-
ILE	232	96	**-2.13**	**-2.59**	**-1.07**	**-1.89**
LYS	233	97	-0.22	-0.28	-	-0.24
ILE	235	99	-0.88	-0.78	-0.47	-0.50
ASN	236	100	**-2.38**	**-2.25**	-0.37	**-2.60**
LYS	237	101	-0.19	-0.16	-0.11	-0.16
ASN	239	103	-0.35	-0.38	-0.18	-0.33
ARG	240	104	-0.17	-	-	-0.11
LYS	241	105	-0.11	-	-	-
LYS	242	106	-0.16	-0.17	-0.20	-0.18
VAL	354	82	-	-	-	-0.22
ASP	356	84	-	-	-0.15	-0.12
PHE	357	85	-0.68	-0.39	**-1.35**	**-1.58**

LEU	358	86	-0.86	-	**-1.56**	**-1.07**
ILE	359	87	-	-	-0.12	-
ALA	361	89	-0.90	-0.71	-0.77	-0.58
PHE	362	90	**-1.35**	-0.16	**-1.85**	-0.16
ILE	364	92	-	-0.89	-	-
PHE	365	93	-0.81	**-1.19**	-0.53	-
ILE	368	96	-	-0.13	-	-
LYS	369	97	-	-0.32	-	-

[*] The corresponding residue ID in single chain of the *E. coli* MscL. .
